# ETSR-YOLO: An improved multi-scale traffic sign detection algorithm based on YOLOv5

**DOI:** 10.1371/journal.pone.0295807

**Published:** 2023-12-14

**Authors:** Haibin Liu, Kui Zhou, Youbing Zhang, Yufeng Zhang

**Affiliations:** 1 Department of Automotive Engineers, Hubei University of Automotive Technology, Shiyan, PR China; 2 Sharing-X Mobile Services Technology Lab, Hubei University of Automotive Technology, Shiyan, PR China; 3 Hubei Provincial Key Laboratory of Automotive Power Transmission and Electronic Control, Shiyan, PR China; VIT-AP Campus, INDIA

## Abstract

In the application of driverless technology, current traffic sign recognition methods are susceptible to the influence of ambient light interference, target size changes and complex backgrounds, resulting in reduced recognition accuracy. To address these challenges, this study introduces an optimisation algorithm called ETSR-YOLO, which is based on the YOLOv5s algorithm. First, this study improves the path aggregation network (PANet) of YOLOv5s to enhance multi-scale feature fusion by generating an additional high-resolution feature layer to improve the recognition of YOLOv5s for small-sized objects. Second, the study introduces two improved C3 modules that aim to suppress background noise interference and enhance the feature extraction capabilities of the network. Finally, the study uses the Wise-IoU (WIoU) function in the post-processing stage to improve the learning ability and robustness of the algorithm to different samples. The experimental results show that ETSR-YOLO improves mAP@0.5 by 6.6% on the Tsinghua-Tencent 100K (TT100K) dataset and by 1.9% on the CSUST Chinese Traffic Sign Detection Benchmark 2021 (CCTSDB2021) dataset. In the experiments conducted on the embedded computing platform, ETSR-YOLO demonstrates a short average inference time, thereby affirming its capability to deliver dependable traffic sign detection for intelligent vehicles operating in real-world traffic scenes. The source code and test results of the models used in this study are available at https://github.com/cbrook16/ETSR-YOLO.

## Introduction

In recent years, the rapid evolution of driverless technology has catalyzed the widespread integration of intelligent vehicles into our transportation systems. Among the various subsystems that constitute intelligent vehicles, the ability to access real-time and dependable traffic information is an imperative prerequisite for effective path planning and decision-making. Within this framework, the traffic sign recognition (TSR) function holds particular importance as it furnishes vehicles with instantaneous and precise road-related data, playing a pivotal role in accident prevention and traffic congestion reduction. TSR systems predominantly rely on optical sensors as their primary signal inputs, which encompass technologies like cameras and LiDAR. Notably, cameras, owing to their cost-effectiveness and capacity to capture richer visual information, emerge as a more suitable choice for versatile traffic sign detection solutions. Furthermore, in regions where smart city infrastructure is not yet extensively developed, TSR systems hold immense potential for further advancements.

Traffic sign detection is the process of identifying the location, class, and size of signs within an image sequence. It traditionally comprises two main phases: detection and classification. During the detection phase, the goal is to pinpoint the signs in the image, while the classification phase assigns the detected signs to their respective categories. Traditional methods have historically relied on either shape-based [1] or color-based [2] techniques due to the distinct color and shape characteristics of traffic signs. In recent years, the development of GPU-accelerated hardware and deep learning techniques has led to state-of-the-art results in object recognition challenges. Methods based on convolutional neural networks (CNN) [3] have overcome the weaknesses of traditional approaches, such as poor robustness and limited applicability. The general process involves extracting candidate regions from an image using a region of interest (ROI) extraction method and then classifying them using a CNN classifier. Common ROI-based algorithms include R-CNN [4], Fast R-CNN [5], Faster R-CNN [6], and Mask R-CNN [7]. In general, the efficiency of these approaches is limited by the performance of the ROI extraction algorithm. Instead of using ROI extraction to obtain candidate regions, the Single Shot Multibox Detector (SSD) [8], RetinaNet [9] and You Only Look Once (YOLO) series use a single neural network structure for simultaneous target localisation and classification, with better real-time performance.

Detecting small objects has long posed a significant challenge in the field of computer vision. Because traffic signs are mostly small targets that occupy a small portion of the image and lack sufficient visual features, they are more difficult to distinguish in real traffic scenes. Moreover, traffic sign detection is highly susceptible to interference from numerous factors, including complex lighting conditions and noise, leading to a considerable reduction in detection accuracy. The emergence of deep learning technologies, especially CNN-based object detection algorithms, has substantially improved the robustness of object recognition. However, the existing methods still have more false detections and omissions when detecting small-sized traffic signs, which cannot meet the reliability requirements in practical applications. Drawing inspiration from the YOLO series of object detection algorithms, this study introduces an enhanced traffic sign detection algorithm named ETSR-YOLO. Experimental results conducted on benchmark datasets illustrate the significant enhancement in small traffic sign detection performance with ETSR-YOLO. These improvements are accompanied by high real-time processing capabilities and robustness, aligning the algorithm with the demands of real-world scenes.

The main contributions of this paper are as follows:

- The Coordinate Attention (CA) [10] mechanism is seamlessly integrated into the backbone network of YOLOv5. This integration serves to effectively suppress noise interference and facilitates the learning of features at critical locations, ultimately enhancing the feature extraction capabilities of the network.
- In the network’s neck section, we have incorporated the ConvNeXt Block [11]. This addition serves to significantly increase the network’s receptive field and minimize the loss of feature information, enhancing the network’s capacity to capture correlations across various spatial locations.
- The Path Aggregation Network (PANet) [12] in YOLOv5 has been augmented to facilitate the extraction of more comprehensive contextual information, thus enhancing the algorithm’s capacity to detect traffic signs of varying sizes.
- To further enhance the algorithm’s performance, we employ the Wise-IoU [13] loss function to refine the predicted bounding boxes in the post-processing stage. The Wise-IoU function incorporates a sophisticated gradient gain allocation strategy and takes into account the degree of outliers. This strategy helps balance bounding box samples of varying quality while mitigating the adverse effects of harmful gradients.

## Related work

Early CNN-based object recognition algorithms often stacked convolutional and fully connected layers, leading to redundant parameters that increased the risk of model overfitting and prolonged training time. To address these issues, Han et al. [14] enhanced the Faster R-CNN for small traffic sign detection by incorporating dilated convolution and eliminating unnecessary network layers. Zhang et al. [15] introduced a cascaded R-CNN structure capable of capturing multi-scale features, improving detection accuracy through cascaded networks and weighted feature refinement. Manzari et al. [16] devised a pyramid transformer, applied to the R-CNN, achieving a remarkable 77.8% mAP on the German Traffic Sign Detection Benchmark (GTSDB). Li et al. [17] addressed the loss of feature information by integrating ResNet50-D and an attention-guided contextual feature pyramid network to enhance the feature extraction capability of Faster R-CNN.

With the introduction of network structures like Spatial Pyramid Pooling (SPP) [18] and Feature Pyramid Network (FPN) [19], the field of multi-scale object detection has seen significant development. Among these advancements, the YOLO series has gained prominence owing to its lightweight architecture and scalability. Avramović et al. [20] improved detection accuracy in automotive applications by combining ROI extraction with various YOLO architectures. Fan et al. [21] enhanced detection speed by adopting DenseNet as the backbone network for YOLOv3. Gong et al. [22] modified YOLOv3’s network header to create 152 × 152 feature maps, improving the detection performance of small traffic signs. Song et al. [23] proposed a Chinese traffic sign detection algorithm that enhances detection accuracy by optimizing the anchor boxes and SPP network of YOLOv4. Wang et al. [24] introduced the improved feature pyramid model AF-FPN to YOLOv5, boosting the detection accuracy of traffic signs in multi-scale scenarios. Shi et al. [25] developed a lightweight small traffic sign detection algorithm to enhance the computational efficiency of YOLOv5. This was achieved by designing a dense neck structure and improving the bounding box (Bbox) regression function. Jia et al. [26] put forth a real-time traffic sign detection algorithm based on YOLOv7. They reduced model complexity by refining the spatial pyramid pooling network and incorporating a weighted attention module to emphasize salient feature regions.

The literature review highlights that much of the research primarily concentrates on enhancing detection accuracy within individual datasets under normal weather conditions. However, there’s a notable gap in comprehensive investigations regarding the impact of factors like light interference and complex environments. The YOLO series comprises popular object recognition algorithms characterized by ongoing and rapid updates. Generally, newer iterations of these algorithms come with optimizations related to performance, speed, and accuracy. Nevertheless, it’s crucial to note that the applicability of various YOLO algorithm versions varies significantly. While YOLOv7 or newer may offer superior real-time performance compared to YOLOv5, it’s not consistently more effective than YOLOv5 in specific application scenarios, particularly when it comes to detecting small objects. Consequently, in selecting the foundational algorithm, this paper opts for the more mature YOLOv5s algorithm. Substantial enhancements focused on traffic sign detection have been applied to address the issues related to the omission or misidentification of traffic signs.

## The proposed method

### The network structure of ETSR-YOLO

**Fig 1:**
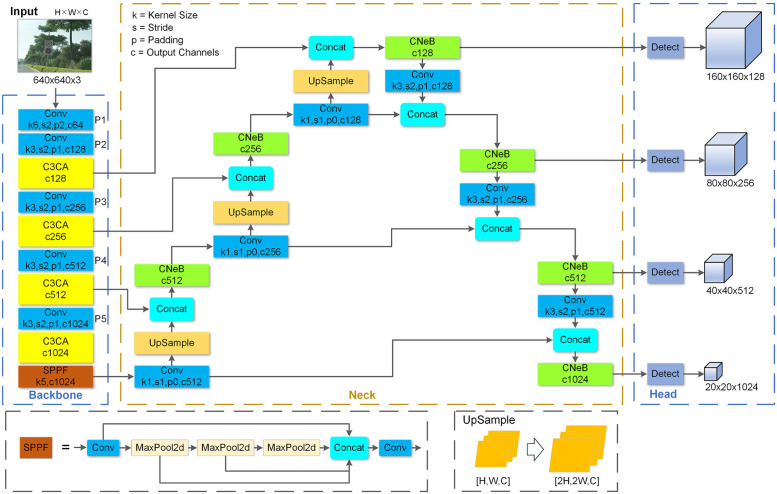
Network structure of ETSR-YOLO.

The overall structure of ETSR-YOLO is shown in Fig 1. The C3CA and CNeB modules with enhanced feature extraction capabilities are used in the ETSR network. By integrating the coordinate attention mechanism and the ConvNeXt Block, the C3CA and CNeB modules can suppress noise interference, increase the receptive field of the network, and reduce the loss of critical feature information. In the neck network, the adapted PANet can fuse four different sizes of feature maps to capture richer contextual information, which helps improve the algorithm’s ability to detect traffic signs of different sizes. Finally, the K-means algorithm is used to recalculate the anchor boxes in the post-processing stage to improve the matching of anchor boxes to small objects. Meanwhile, the Wise-IoU loss function is used to further improve the localisation accuracy of the Bbox. Overall, the improved network has better detection capability and robustness to traffic signs of different sizes.

### Improvements to the C3 module

The C3 module of YOLOv5 is proficient at extracting object features. However, the simple structure of the bottleneck layer within the C3 module results in YOLOv5’s weak detection of small targets and requires more training data to ensure the model’s ability to generalise. To address this, we extended the C3 module to incorporate CA mechanisms and the ConvNeXt Block.

**Fig 2:**
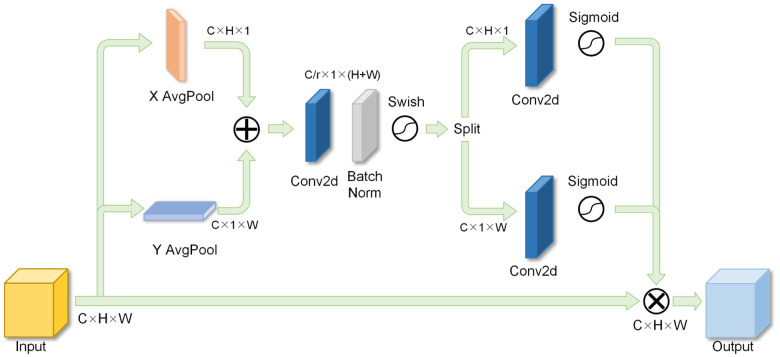
Structure of coordinate attention.

The attention mechanism effectively mitigates background noise interference by directing its focus towards the salient feature regions of objects, resulting in the generation of higher-quality feature maps. Conventional attention mechanisms, such as the Squeeze-and-Excitation Network (SENet) [27] and the Convolutional Block Attention Module (CBAM) [28], excel at extracting critical features from feature channels or spatial regions. However, these methods can only capture local information about the object and cannot capture long-range dependencies. In contrast, the CA mechanism introduces positional awareness by considering both feature channels and positional information. This enables the network to concentrate on a broader pixel area, facilitating the more precise localization of sensitive regions. The structure of CA is shown in Fig 2.

The CA structure comprises two main components: coordinate information embedding and coordinate attention generation, aimed at establishing long-range dependencies within the target. In contrast to the conventional approach of utilizing 2D global pooling for channel attention generation, the coordinate attention mechanism divides the 2D global pooling process into two distinct 1D encoding processes. These processes independently encode input features along the horizontal and vertical directions, thereby acquiring X-axis and Y-axis positional information. The equations for computing the coordinate information are presented in Eqs 1 and 2.

$$\begin{array}{c} z_{c}^{h}\left(h\right)=\frac{1}{W}\sum_{0\leq i<W}I_{c}\left(h,i\right) \end{array}$$ (1)

$$\begin{array}{c} z_{c}^{w}\left(w\right)=\frac{1}{H}\sum_{0\leq j<H}I_{c}\left(j,w\right) \end{array}$$ (2)

where $z_{c}^{h}\left(h\right)$ is a perceptual feature in the vertical direction and $z_{c}^{w}\left(w\right)$ is a perceptual feature in the horizontal direction. *W* and *H* are the width and height of the feature map and *I*_*c*_ represents the input features.

Once the coordinate information is embedded, the coordinate attention graph can be constructed. Initially, the perceptual features in both directions are concatenated, followed by a transformation via a 1×1 convolution operation to derive the relation map *f*_*c*_, as demonstrated in Eq 3.

$$\begin{array}{c} f_{c}={\text{Conv}}^{1x1}\left(\text{concat}\left(z_{c}^{h},z_{c}^{w}\right)\right) \end{array}$$ (3)

Following the acquisition of the relation map, it undergoes slicing, transposition, convolution, and activation to yield a pair of location-sensitive attention weights denoted as $g_{c}^{h}$ and $g_{c}^{w}$, as depicted in Eqs 4 and 5.

$$\begin{array}{c} g_{c}^{h}=\sigma \left({\text{Conv}}^{1x1}\left(f_{c}^{h}\right)\right) \end{array}$$ (4)

$$\begin{array}{c} g_{c}^{w}=\sigma \left({\text{Conv}}^{1x1}\left(f_{c}^{w}\right)\right) \end{array}$$ (5)

By applying these weights to the inputs *I*_*c*_, we obtain the modified feature map *O*_*c*_, as demonstrated in Eq 6.

$$\begin{array}{c} O_{c}\left(i,j\right)=I_{c}\left(i,j\right)\times g_{c}^{h}\left(i\right)\times g_{c}^{w}\left(j\right) \end{array}$$ (6)

**Fig 3:**
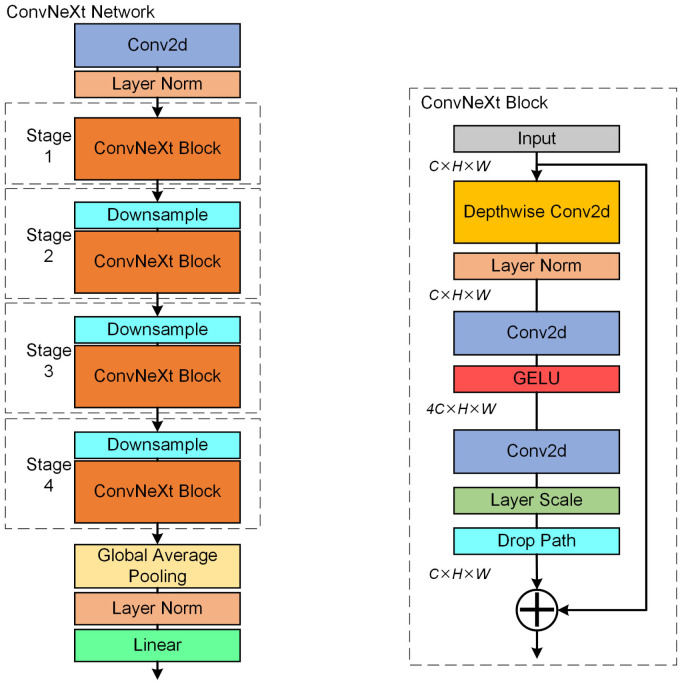
Network structure of ConvNeXt and ConvNeXt Block.

In recent years, computer vision research has witnessed the emergence of vision transformers (ViT) [29] equipped with self-attention capabilities. Among these, the Swin-Transformer [30] has gained popularity, boasting a hierarchically designed network architecture that employs a sliding window approach to aggregate feature information across windows, thus expanding its visual receptive field. In contrast to ViT’s structure, ConvNeXt is a CNN model that incorporates Swin-Transformer’s optimization strategies to enhance both computational efficiency and model accuracy. The network structure of ConvNeXt and ConvNeXt Block is shown in Fig 3.

The ConvNeXt network is divided into four stages, each consisting of a downsampling layer and a ConvNeXt Block. As the input features pass through, their receptive fields are continuously expanded. The ConvNeXt Block incorporates various enhancement strategies, including depthwise separable convolution (DSC) [31], an inverted bottleneck architecture, layer normalization (LN) [32], and the utilization of Gaussian Error Linear Units (GELU) [33] as activation functions. In the context of transformers, the residual link plays a vital role in constructing both the encoder and decoder. The ConvNeXt Block also leverages the residual link but introduces DSC in the initial step and enlarges the convolution kernel size from 3×3 to 7×7.

**Fig 4:**
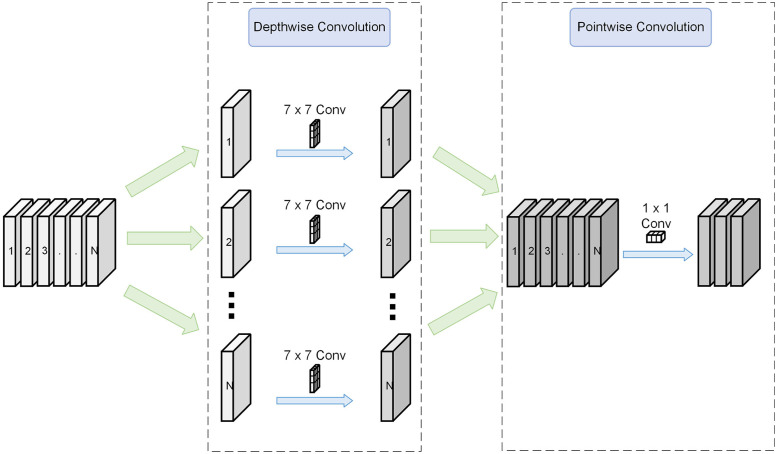
Composition of the depthwise separable convolution.

As depicted in Fig 4, the DSC structure comprises two components: depthwise convolution and pointwise convolution. In DSC, individual convolution kernels are employed for each channel of the feature map, followed by pointwise convolution on the merged feature map. This design makes DSC computationally efficient, making it particularly suitable for embedded platforms with constrained computing power.

**Fig 5:**
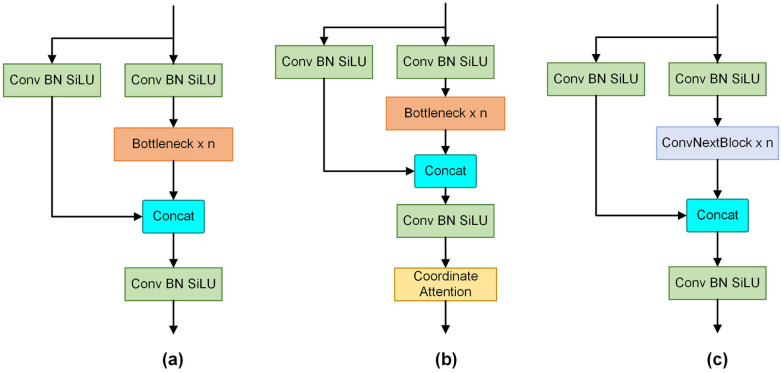
Block designs for the original C3 module, the C3CA module, and the CNeB module. (a) is the structure of the original C3. (b) is the structure of the C3CA. (c) is the structure of the CNeB.

To create the C3CA and CNeB modules, we integrate CA and ConvNeXt Block into the outputs of the C3 module, as illustrated in Fig 5. This approach avoids the need to introduce extra network layers and proves to be computationally efficient compared to inserting these modules separately into the network.

### Improvement of the loss function

The regression loss function is an important component of object detection algorithms. It is used to measure the difference between the predicted results and the ground truth annotation. Numerous regression loss functions are rooted in Intersection over Union (IoU), including well-known variants like GIoU [34], DIoU [35], and CIoU [35], each characterized by distinct functional designs. However, recent research efforts have predominantly focused on enhancing the fitting of Bbox loss and emphasizing regression on low-quality examples. This approach introduces redundant computations and impedes potential advancements in algorithm performance.

In YOLOv5, the CIoU function is used for Bbox regression loss calculation. Although CIoU takes into account the intersection area, centroid distance and the aspect ratio of the Bboxes, it is computationally complex and some cases cannot be evaluated, which can adversely affect the evaluation of the sample. EIoU [36] simplifies this by eliminating the need to calculate aspect ratio, allowing direct application of the penalty factor to the prediction box. This improves regression accuracy and convergence speed. Focal-EIOU [36] goes a step further to address sample quality imbalance. However, it employs static focusing and may not fully exploit the potential of its non-monotonic focusing mechanism. When dealing with datasets containing low-quality samples, over-focusing regression on these samples can degrade model performance.

In this paper, we replace the default CIoU function with the more effective Wise-IoU (WIoU) loss function. WIoU is a dynamic, non-monotonically focused loss function that incorporates an outlier degree and an improved gradient gain allocation strategy for Bbox regression. It balances attention across samples of varying quality, reduces the generation of harmful gradients, and enhances overall detector performance. WIoU comes in three versions: WIoUv1, WIoUv2, and WIoUv3. WIoUv1, for example, constructs an attention-based Bbox loss using distance metrics, aiding convergence, model generalization, and reducing emphasis on centroid distance when prediction boxes overlap well with target boxes. The equation for WIoUv1 is shown in Eqs 7 and 8.

$$\begin{array}{c} L_{WIoUv1}=R_{WIoU}L_{IoU} \end{array}$$ (7)

$$\begin{array}{c} R_{WIoU}=\text{exp}\left(\frac{{\left(x-x^{gt}\right)}^{2}+{\left(y-y^{gt}\right)}^{2}}{{\left({\left(w^{c}\right)}^{2}+{\left(h^{c}\right)}^{2}\right)}^{*}}\right) \end{array}$$ (8)

where *L*_*IoU*_ ∈ [0, 1] and *R*_*WIoU*_ ∈ [1, *e*). *R*_*WIoU*_ is the distance metric and *W*_*c*_ and *H*_*c*_ are the width and height of the smallest enclosing box between the two Bboxes. To prevent *R*_*WIoU*_ from generating gradients that prevent convergence, *W*_*c*_ and *H*_*c*_ are separated from the computational graph and are not needed for backpropagation of its gradients.

WIoUv2 introduces a Focal Loss-inspired monotonic focusing mechanism, which balances the contribution of samples with varying difficulty to improve classification performance. The equation for WIoUv2 is given in Eq 9.

$$\begin{array}{c} L_{WIoUv2}=L_{IoU}^{\gamma *}L_{WIoUv1},\gamma >0 \end{array}$$ (9)

where $L_{IoU}^{\gamma *}$ is the monotonic focusing coefficient.

In WIoUv3, the quality of the Bbox is defined by the degree of outliers. It assigns small gradient gains to high-quality Bboxes with minimal outliers and lower gradient gains to low-quality Bboxes with significant outliers. This reduction in harmful gradients from low-quality examples enables WIoUv3 to adapt its gradient gain allocation strategy to the current situation. The equation for WIoUv3 is shown in Eqs (10–12).

$$\begin{array}{c} L_{WIoUv3}=rL_{WIoUv1} \end{array}$$ (10)

$$\begin{array}{c} r=\frac{\beta }{\delta \alpha^{\beta -\delta }} \end{array}$$ (11)

$$\begin{array}{c} \beta =\frac{L_{IoU}^{*}}{\bar{L_{IoU}}}\in [0,+\infty ) \end{array}$$ (12)

where *r* is the non-monotonic focusing coefficient, *β* is the degree of Bbox quality outlier and $\bar{L_{IoU}}$ is the moving average with momentum value *m*.

### Enhanced multi-scale feature fusion

**Fig 6:**
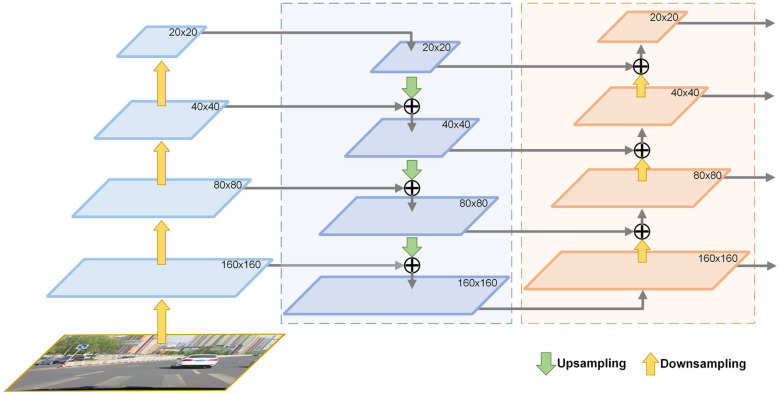
Enhanced path aggregation network.

In YOLOv5, PANet is used to fuse feature maps of different sizes from the backbone network. However, by default, this fusion process results in feature maps of sizes 80×80, 40×40, and 20×20. Smaller feature maps capture fewer details of objects, which isn’t ideal for detecting small objects like traffic signs. In our approach, we deepen the PANet network to generate larger 160×160 feature maps. As illustrated in Fig 6, we incorporate an upsampling layer into the neck network, creating 160×160 feature maps that are concatenated with feature maps of the same size and channel count in the backbone network. Additionally, to maximize the utilization of these larger feature maps, we downsample them once to enhance the expressiveness of the smaller feature maps. This results in feature maps of four different sizes (160×160, 80×80, 40×40, and 20×20) for detecting traffic signs of various scales, from the smallest to the largest. This approach significantly improves the detection of small traffic signs without substantially increasing computational complexity or compromising the accuracy of detecting larger signs. With the introduction of these new detection layers, the default anchor boxes are no longer suitable. Therefore, we utilize YOLOv5’s K-means clustering function to generate anchor boxes tailored to our modified detection headers.

## Experiments and discussions

### Experimental environment

The hardware and software platforms used for model training are listed in Table 1.

**Table 1:** The hardware and software environment used for the experiment.

Item	Version
CPU	Intel(R) Core(TM) i7–10870H Processor
GPU	NVIDIA RTX 2080Ti
Operating System	Ubuntu 22.04
Python	3.8.15
PyTorch	1.8.2
CUDA	11.1
cuDNN	8.0.5

Table 2 presents the hyperparameters used during model training. The neural network undergoes multiple iterations on the dataset images to obtain the optimal weights.

**Table 2:** Model training parameters.

Attribute	Value
Input Image Size	[640, 640]
Initial Learning Rate	0.01
Batch Size	32
Epoch	200
Early Stopping	50
Weight Decay	0.0005
Momentum	0.937
Mosaic Enhancement	True
Mixup	True
Optimizer	Stochastic Gradient Descent

This study utilized the TT100K (Tsinghua-Tencent 100K) dataset [37] and the CCTSDB2021 (CSUST Chinese Traffic Sign Detection Benchmark 2021) dataset [38] for model training and validation. TT100K is a Chinese traffic sign recognition benchmark dataset with a resolution of 2048×2048 per image, encompassing 128 labelled traffic sign categories. To improve training efficiency, 45 categories with more than 100 samples each were selected, resulting in 8,293 images. Among these, 6,634 were designated for training, and 1,659 for testing. The CCTSDB2021 dataset consists of 17,856 images captured on roads and highways in various Chinese cities, featuring different lighting and weather conditions. It comprises three common categories of traffic signs: warning, prohibitory, and mandatory. For this study, 14,285 images were used for training and 3,571 images for testing.

**Fig 7:**
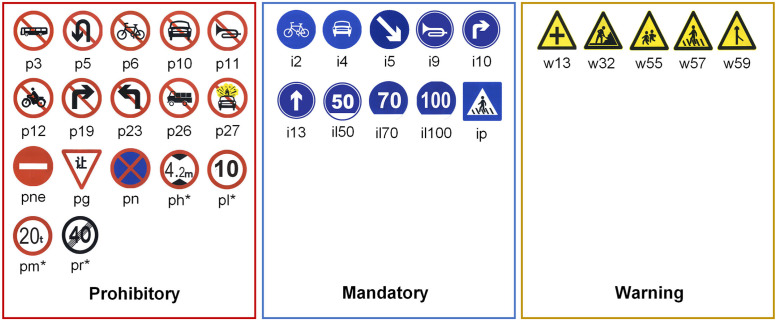
Visualisation of different categories of traffic signs.

**Fig 8:**
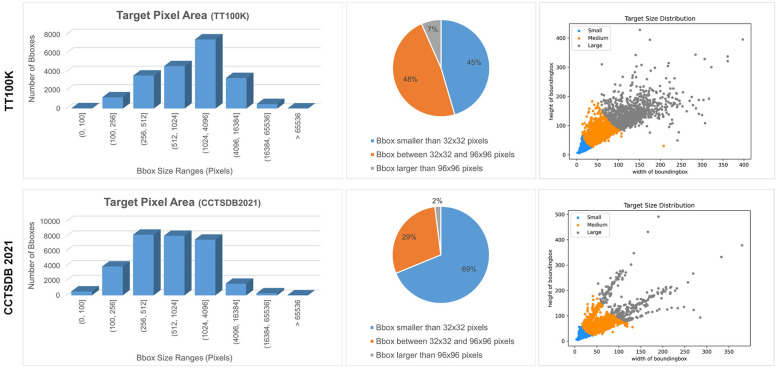
Statistical visualisation of the TT100K and CCTSDB2021 datasets.

Fig 7 displays visualizations of different traffic sign categories, while Fig 8 presents statistical information regarding the two datasets. The analysis indicates that the TT100K dataset exhibits a relatively balanced distribution of signs, primarily falling within medium (pixel area between 32^2^ and 96^2^) and large sizes (pixel area > 96^2^). In contrast, the CCTSDB2021 dataset contains more small signs (pixel area < 32^2^) and shows greater variability in sign sizes.

### Evaluation index

Several common metrics are used to evaluate model performance, including precision (P), recall (R), F1, mean average precision (mAP), number of parameters (Params), frames per second (FPS), and model weight file size (Size). Precision (P) measures the algorithm’s accuracy in detecting a single category, while mAP assesses multi-category object detection accuracy. The number of parameters, model file size, and FPS gauge the model’s complexity and detection speed. The equations for P, R, F1, AP, and mAP are provided in Eqs (13–17).

$$\begin{array}{c} P=\frac{TP}{TP+FP} \end{array}$$ (13)

$$\begin{array}{c} R=\frac{TP}{TP+FN} \end{array}$$ (14)

$$\begin{array}{c} F1=2\frac{PR}{P+R} \end{array}$$ (15)

$$\begin{array}{c} AP=\int_{0}^{1}P(R)d(R) \end{array}$$ (16)

$$\begin{array}{c} mAP=\frac{\sum_{i=1}^{N}\;AP_{i}}{N} \end{array}$$ (17)

### Analysis of experimental results on the TT100K dataset

#### Ablation experiments

In this section, we analyze the impact of various enhancement methods on the performance of the YOLOv5s algorithm through ablation experiments using the TT100K dataset. The results are summarized in Table 3.

**Table 3:** Ablation experiments.

No.	Enhanced Feature Fusion Network	CA	CNeB	WIoU	P(%)	R(%)	F1(%)	mAP@0.5(%)	Params(M)
1					83.9	77.4	80.5	81.7	7.1
2	✓				89.0 (+5.1)	82.5 (+5.1)	85.6 (+5.1)	87.2 (+5.5)	7.3 (+0.2)
3	✓	✓			89.7 (+5.8)	82.8 (+5.4)	86.1 (+5.6)	87.8 (+6.1)	7.3 (+0.2)
4	✓	✓	✓		87.4 (+3.5)	82.5 (+5.1)	84.8 (+4.3)	87.9 (+6.2)	7.5 (+0.4)
5	✓	✓	✓	v1	86.0 (+2.1)	82.7 (+5.3)	84.3 (+3.8)	86.4 (+4.7)	7.5 (+0.4)
6	✓	✓	✓	v2	88.0 (+4.1)	82.8 (+5.4)	85.3 (+4.8)	87.5 (+5.8)	7.5 (+0.4)
7	✓	✓	✓	v3	88.9 (+5.0)	83.6 (+6.2)	86.1 (+5.6)	88.3 (+6.6)	7.5 (+0.4)

Table 3 demonstrates the effectiveness of various enhancements, including the enhanced feature fusion network, CA, CNeB, and WIoU, in improving the F1 and mAP of the model. Notably, integrating the small object detection layer into the YOLOv5s model significantly improved detection accuracy, resulting in a 5.1% increase in F1 and a 5.5% increase in mAP@0.5. This enhancement particularly benefits the detection of small traffic signs, enhancing overall model performance without significantly increasing computational cost. The combination of the enhanced feature fusion network and the coordinate attention mechanism improved F1 by 0.5% and mAP@0.5 by 0.6%, emphasizing CA’s role in capturing object details. Replacing the C3 module with the CNeB module at the neck showed a slight decrease in precision and recall, a 1.3% decrease in F1, and a marginal increase in parameters. However, mAP continued to increase by approximately 0.1%, indicating that CNeB can replace the bottleneck layer for feature extraction. Additionally, we tested three versions of WIoU, with WIoUv3 providing the most significant improvement, achieving an F1 of 86.1% and a mAP of 88.3%. This suggests that WIoUv3’s outlier degree and gradient gain allocation strategy improves Bbox quality and reduces harmful gradients, making it more effective for traffic sign detection. Despite these improvements slightly increasing model complexity and the number of parameters, the model remains capable of real-time detection and is suitable for deployment on mobile platforms.

**Fig 9:**
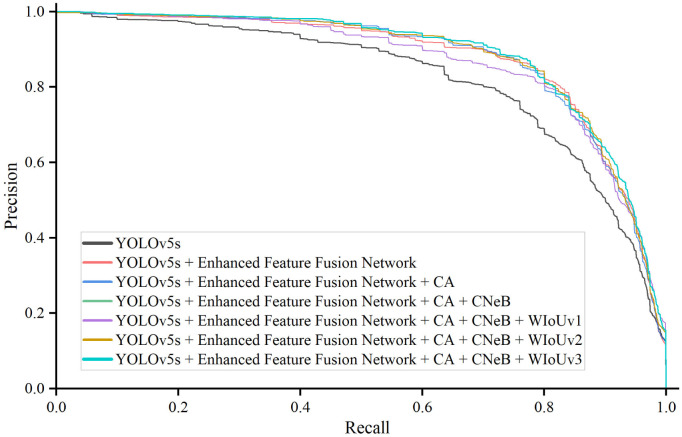
Precision-recall curve for improved models.

Fig 9 shows the precision-recall curves for each improved model. The modifications made to YOLOv5s had a positive impact on both precision and recall, resulting in a wider range of curves. The integration of the enhanced feature fusion network significantly improves the overall performance, while the addition of CA and CNeB leads to slight fluctuations in precision and recall. When comparing models incorporating three different versions of WIoU, WIoUv3 outperforms the others. Consequently, we refer to the model using WIoUv3 as ETSR-YOLO. More detailed precision and recall data can be found in S1 Data.

#### Performance comparison with other models

We conducted training using several well-known detection models for performance comparison on the TT100K dataset. These models include YOLOv3, YOLOX, YOLOv6, YOLOv7, DAMO-YOLO, and the classic two-stage algorithm Faster R-CNN. A summary of the training results is presented in Table 4.

**Table 4:** Performance comparison with popular detection models on the TT100K dataset.

Model	Input(Pixels)	mAP@0.5(%)	mAP@0.5:0.95(%)			Params(M)	Speed(FPS)
			Small	Medium	Large		
Faster R-CNN [6]	600×600	55.1	21.7	46.4	59.3	41.6	15
YOLOv3 [39]	416×416	81.2	46.9	68.7	72.2	61.7	31
YOLOv5s	640×640	81.7	48.3	69.7	70.2	7.1	118
YOLOv5m	640×640	83.1	45.7	73.6	81.0	21.0	57
YOLOv5l	640×640	84.8	52.0	72.8	75.0	46.3	42
YOLOX [40]	640×640	87.1	53.8	73.4	75.7	54.2	28
YOLOv6 [41]	640×640	83.4	46.8	73.5	82.6	18.5	53
YOLOv7 [42]	640×640	85.3	44.7	73.3	75.2	37.4	44
YOLOv7-Tiny [42]	640×640	76.5	37.4	69.1	76.8	6.1	134
DAMO-YOLO [43]	640×640	84.8	42.5	76.7	84.3	15.9	36
ETSR-YOLO	640×640	88.3	54.9	73.8	76.6	7.5	88

Table 4 demonstrates that ETSR-YOLO achieves the highest accuracy, excels in detecting small targets, maintains a low parameter count, and exhibits a clear advantage in detection speed. Notably, compared to models with more parameters, such as Faster R-CNN, YOLOv3, YOLOv5l, and YOLOX, ETSR-YOLO attains the highest mAP@0.5. To evaluate the model’s performance across different-sized traffic signs, we computed mAP@0.5:0.95 for objects of various sizes following the Microsoft COCO standard. For detecting small targets, the improved model’s mAP@0.5:0.95 outperforms YOLOv3 by 8%, YOLOv5l by 2.9%, and YOLOX by 1.1%. In comparison to newer models like YOLOv6, YOLOv7, and DAMO-YOLO, which are more inclined towards medium and large targets, ETSR-YOLO displays a significant advantage in detecting small targets while also offering superior parameters and detection speed. When compared to the pre-improved YOLOv5s model, ETSR-YOLO enhances mAP@0.5:0.95 by 6.6% for small targets, 4.1% for medium targets, and 6.4% for large targets. Despite the increased complexity, the improved model maintains a real-time detection speed of 88 FPS, indicating that the enhancements in this paper do not significantly impact the algorithm’s real-time performance.

**Fig 10:**
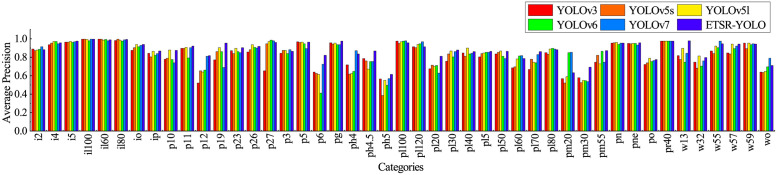
Average precision of YOLOv3, YOLOv5, YOLOv6, YOLOv7 and ETSR-YOLO in each category.

We collected average precision data for YOLOv3, YOLOv5s, YOLOv5l, YOLOv6, YOLOv7, and ETSR-YOLO on each category within the validation dataset. The visualisation of the data is shown in Fig 10. Compared to YOLOv5s, ETSR-YOLO significantly improves the detection accuracy for most categories of traffic signs. Furthermore, ETSR-YOLO demonstrates comparable or even superior detection accuracy to more complex models, highlighting its unique advantages.

**Fig 11:**
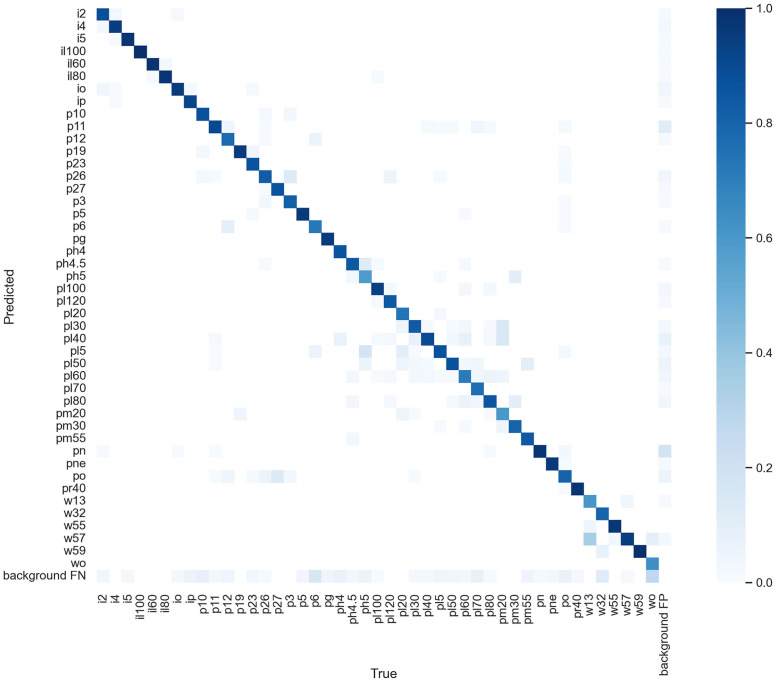
Confusion matrix of ETSR-YOLO.

The confusion matrix for each category in ETSR-YOLO is depicted in Fig 11. Notably, the prediction results predominantly align with the diagonal of the confusion matrix, displaying lighter colors elsewhere. This signifies ETSR-YOLO’s robust ability to distinguish among multiple categories, resulting in reliable classification accuracy.

To further assess our model’s performance, we provide a comparison with recently developed detection models. The performance results of various models are presented in Table 5.

**Table 5:** Performance comparison of the improved network models on the TT100K dataset.

Model	Input(Pixels)	mAP@0.5(%)	Params(M)	Size(MB)	Speed(FPS)	GPU
SC-YOLO [25]	640×640	90.4	6.1	/	33.7	3080
Improved YOLOv4 [44]	416×416	87.3	/	16.0	70.5	1660Ti
GCM-YOLOv5 [45]	640×640	83.1	5.2	10.9	/	2060
YOLO-SG [46]	640×640	75.8	4.0	8.8	131.6	V100
AIE-YOLO [47]	640×640	84.8	7.9	/	100.7	3090
MSA-YOLOv3 [48]	544×544	86.3	/	/	23.8	P100
ETSR-YOLO	640×640	88.2	7.5	15.3	88.0	2080Ti

The proposed model in this paper achieves high detection accuracy, outperforming many state-of-the-art models. While there’s a 2.2% difference in mAP compared to SC-YOLO, our model demonstrates excellent real-time performance on a 2080Ti GPU due to its higher FPS. Overall, our model excels in accuracy and speed on the TT100K dataset.

### Analysis of experimental results on the CCTSDB2021 dataset

#### Performance comparison with other models

We conducted experiments on the CCTSDB2021 dataset using the same network model and training strategy, and the results are summarized in Table 6.

**Table 6:** Performance comparison with popular detection models on the CCTSDB2021 dataset.

Model	Input(Pixels)	mAP@0.5(%)	mAP@0.5:0.95(%)			Params(M)	Speed(FPS)
			Small	Medium	Large		
Faster R-CNN [6]	600×600	74.6	25.4	61.2	74.4	41.6	21
YOLOv3 [39]	416×416	97.4	68.9	82.4	90.3	61.7	42
YOLOv5s	640×640	96.4	69.7	85.4	97.6	7.1	156
YOLOv5m	640×640	97.3	73.4	88.5	98.4	21.0	78
YOLOv5l	640×640	97.3	74.6	89.2	98.9	46.3	56
YOLOX [40]	640×640	95.2	50.4	74.0	90.5	54.2	39
YOLOv6 [41]	640×640	96.9	67.8	83.8	95.6	18.5	70
YOLOv7 [42]	640×640	93.0	60.5	78.9	92.8	37.4	58
YOLOv7-Tiny [42]	640×640	73.8	35.8	67.2	77.9	6.1	181
DAMO-YOLO [43]	640×640	97.1	71.1	85.7	95.9	15.9	45
ETSR-YOLO	640×640	98.3	79.5	93.4	99.3	7.5	108

Table 6 demonstrates that ETSR-YOLO performs admirably on the CCTSDB2021 dataset, surpassing many popular models. Compared to the pre-improved YOLOv5s model, ETSR-YOLO enhances mAP@0.5 by 1.9%, with notable improvements of 9.8% for small targets, 8.0% for medium targets, and 1.7% for large targets. These results highlight the model’s effectiveness in addressing misdetection or omission issues and its suitability for various traffic sign sizes.

Table 7 lists some of the recent traffic sign detection studies to further validate the effectiveness of ETSR-YOLO. ETSR-YOLO achieves a superior mAP compared to the current state-of-the-art models on the CCTSDB2021 dataset. Nonetheless, there is potential for further enhancements in ETSR-YOLO’s detection speed and model complexity.

**Table 7:** Performance comparison of the improved network models on the CCTSDB2021 dataset.

Model	Input(Pixels)	mAP@0.5(%)	Params(M)	Size(MB)	Speed(FPS)	GPU
TSR-YOLO [23]	416×416	92.7	/	41.3	80.5	2060
SC-YOLO [25]	640×640	84.3	6.1	/	/	3080
Improved YOLOv4 [44]	416×416	88.7	/	9.0	208.0	1660Ti
Improved YOLOv3 [49]	640×640	86.1	48.3	/	178.5	2080Ti
Improved YOLOv4 [50]	608×608	90.4	/	/	25.3	3090
M-YOLO [51]	416×416	97.8	/	/	84.0	2080Ti
T-YOLO [52]	608×608	97.3	/	/	19.0	2080Ti
ETSR-YOLO	640×640	98.3	7.0	14.2	108.0	2080Ti

#### Performance comparison in different scenarios

**Fig 12:**
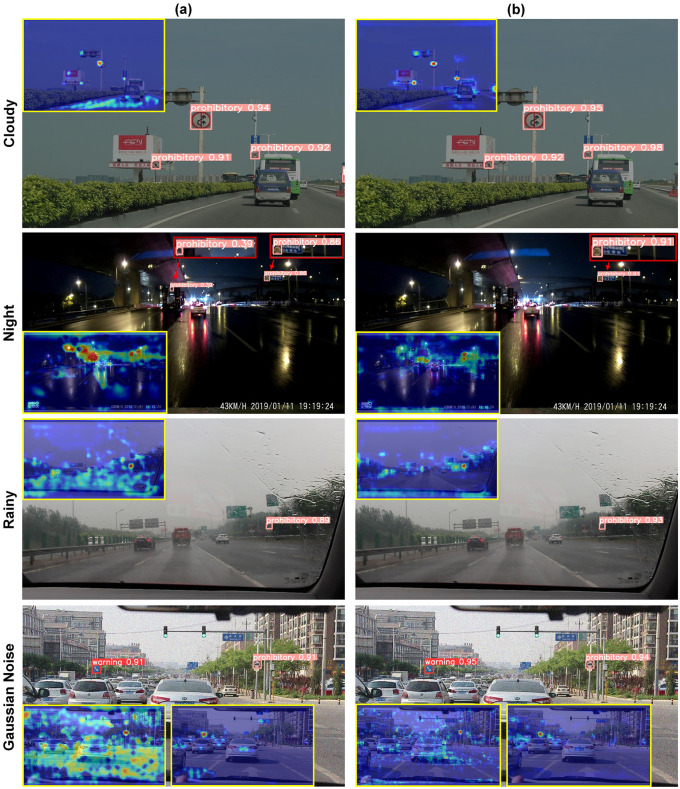
Sample test results using YOLOv5s and ETSR-YOLO in different scenarios. (a) is the test results of YOLOv5s. (b) is the test results of ETSR-YOLO.

We compared the test results of YOLOv5s and ETSR-YOLO under various scenarios, as illustrated in Fig 12. These scenarios included cloudy, nighttime, rainy, and Gaussian noise conditions, enabling us to analyze how the models perform when traffic signs are disrupted. To visually analyze the thermal distribution of feature maps and critical areas, we employed Grad-CAM [53]. Heat maps were generated from the neck network’s output. We identified samples with the target, highlighted them with a yellow border, and placed them in the test result image.

These images demonstrate the significant impact of lighting variations and noise on feature quality. Notably, YOLOv5s exhibits lower performance in detecting small signs, resulting in missed and false detections. The heatmap reveals that YOLOv5s shows a more scattered distribution of hotspots, with some clustering in non-critical areas. In contrast, ETSR-YOLO achieves higher scores, with hotspots concentrated in the target region, indicating less interference over a larger pixel area. In summary, ETSR-YOLO effectively filters out invalid feature regions and demonstrates superior feature extraction capabilities, making it better suited for traffic sign detection.

### Experiments in road scenes

**Fig 13:**
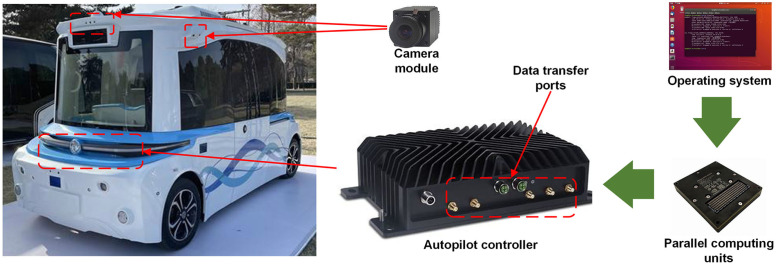
External experimental platform.

To further validate the robustness of ETSR-YOLO under continuous driving conditions, we captured real road scenes using external cameras and deployed the model and sample data in an embedded mobile platform for validation. Fig 13 shows the controller unit used in this experiment, which contains multiple NVIDIA Jetson AGX Xavier processing units to enable mobile parallel computation of neural network models. To conduct the experiments, the sample scenes are manually collected by driving a collection vehicle that captures the city streets through fixed cameras, and the collected samples are used to evaluate the detection performance of the model offline.

For the computing unit, we used the same software environment as for the desktop platform. To reduce the false positive rate, where the confidence threshold is set to 0.6, the NMS IoU threshold is set to 0.45 and the rest of the parameters remain unchanged. Table 8 shows the average inference speed on the platform for the models before and after the improvement. The data shows that the inference speed of the model is not as good as the desktop platform in environments with limited computing power. At the same time, the improved methods in this paper generate additional computations that slightly increase the inference time.

**Table 8:** Comparison of inference speed between YOLOv5s and ETSR-YOLO on the Jetson AGX Xavier platform.

Model	Pre-Process	Inference	Non-Maximum Suppression
YOLOv5s	1.2ms	32.2ms	1.4ms
ETSR-YOLO	1.1ms	38.8ms	1.9ms

**Fig 14:**
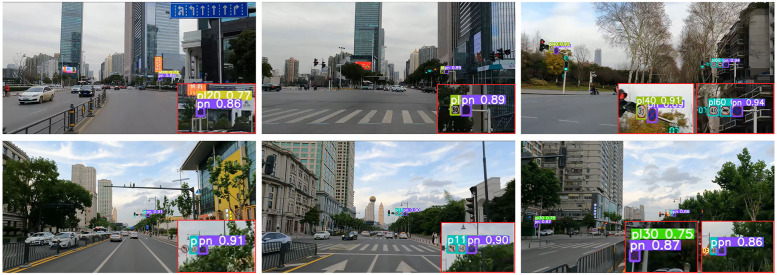
Detection results on sample sequence using ETSR-YOLO.

Fig 14 shows the detection effect of ETSR-YOLO on sample sequences. It can be seen that the improved model performs well.

### Discussion of the model

In this paper, the network structure of YOLOv5 is improved for the characteristics of traffic signs in images. According to the experimental data, it can be seen that the improved method proposed in this paper shows high accuracy in complex road scenes, but there are still some shortcomings:

First, the improved model in this paper cannot effectively improve the accuracy of each detection category. Experiments on the TT100K dataset show that the average accuracy of the improved model is not good enough for some categories, such as ‘ph5’, ‘pm20’ and ‘wo’. This indicates that the network constructed with the preset parameters still has difficulties in recognising certain categories. In this respect, the network parameters can be appropriately increased to improve the recognition accuracy of complex categories at the expense of some of the network inference speed.

In addition, the model does not allow the detection of multi-scale targets to reach a balanced state: although ETSR-YOLO can detect targets from four different scales, its detection accuracy for small traffic signs is not good enough. The main reason for this situation is that the pixel features of small targets are very weak and can easily interfere with other parts of the image. Therefore, better solutions need to be explored to further improve the detection of multi-scale targets.

Finally, the experiments carried out on the Jetson AGX Xavier platform highlight the problems with our model. The improved model is not able to achieve comparable real-time results on the embedded platform as on the desktop platform. We also found that the model sometimes misidentified parts of the surrounding buildings as traffic signs. This type of misidentification problem is more common in complex scenes. Future development plans will further optimise the real-time and robustness of the model on the embedded platform. More attention will also be paid to the quality of the training dataset to improve the richness of the samples.

## Conclusion

In real traffic scenes, deep learning-based traffic sign recognition algorithms must be optimized to ensure real-time and reliable detection. This paper introduces ETSR-YOLO, a novel algorithm designed to address traffic sign recognition challenges in road scenes.

In this paper, we propose several improvements to YOLOv5 for traffic sign detection. First, we enhance the path aggregation network to capture richer contextual information, which in turn improves the detection of traffic signs of different sizes. Second, we embed the coordinate attention mechanism in the backbone network to adaptively enhance important features and suppress noise. Third, we integrate the ConvNeXt block to expand the receptive field of the network and reduce information loss during feature fusion. Finally, we use the WIoU function in post-processing to improve the predictability and robustness of the model. Experimental results demonstrate the effectiveness of our approach. On the TT100K dataset, our model improves mAP@0.5 by 6.6% and achieves a recognition speed of 88 FPS. On the CCTSDB2021 dataset, mAP@0.5 improves by 1.9% with a recognition speed of 108 FPS, outperforming other models. In experiments conducted on the Jetson AGX Xavier platform, ETSR-YOLO exhibits a short average inference time, affirming its capability to deliver reliable real-time detection. Future research will focus on optimising the model’s performance in complex road environments and improving computational efficiency for more reliable traffic sign recognition on in-vehicle embedded platforms.

## Supporting information

S1 Data Precision-Recall data for improved models. (XLSX)
